# First Molecular Insights into the Presence of Canine Kobuvirus in Ecuadorian Dogs Through the Standardization of a Sensitive SYBR Green RT-qPCR Assay

**DOI:** 10.3390/vetsci12111076

**Published:** 2025-11-10

**Authors:** Camila Sanchez-Castro, Anthony Loor-Giler, Silvana Santander-Parra, Martín Campos, Renán Mena-Pérez, Santiago Prado-Chiriboga, Luis Nuñez

**Affiliations:** 1 Facultad de Ingeniería y Ciencias Aplicadas, Carrera de Ingeniería en Biotecnología, Universidad de Las Américas (UDLA), Antigua Vía a Nayón S/N, Quito EC 170124, Ecuador; camila.sanchez.castro285@hotmail.com; 2 Laboratorios de Investigación, Dirección General de Investigación, Universidad de Las Américas (UDLA), Antigua Vía a Nayón S/N, Quito EC 170124, Ecuador; a.abel.loor.giler@gmail.com; 3 Facultad de Ciencias Veterinarias, Universidad de Buenos Aires, Av. Chorroarín 280, Buenos Aires 1427, Argentina; 4 Facultad de Ciencias de la Salud, Carrera de Medicina Veterinaria, Universidad de Las Américas (UDLA), Antigua Vía a Nayón S/N, Quito EC 170124, Ecuador; silvanahsp@yahoo.com (S.S.-P.); sdpch2021@gmail.com (S.P.-C.); 5 Facultad de Industrias Agropecuarias y Ciencias Ambientales, Carrera Medicina Veterinaria, Universidad Politécnica Estatal del Carchi (UPEC), Antisana S/N y Av. Universitaria, Tulcán EC 040102, Ecuador; rolando.campos@upec.edu.ec; 6 Facultad de Ciencias Veterinarias, Universidad Nacional de Rosario (UNR), Boulevard Ovidio Lagos y Ruta 33 Casilda, Santa Fe 2170, Argentina; 7 Facultad de Medicina Veterinaria y Zootecnia, Universidad Central del Ecuador, Gatto Sobral y Jerónimo Leiton, Quito EC 170521, Ecuador; rpmena@uce.edu.ec; 8 Clínica Veterinaria Docente, Universidad de Las Américas (UDLA), Calle Shuara N40-55y Av. de Los Granados, Quito EC 170503, Ecuador; 9 One Health Research Group, Facultad de Ciencias de la Salud, Universidad de Las Américas (UDLA), Antigua Vía a Nayón S/N, Quito EC 170124, Ecuador

**Keywords:** Canine Kobuvirus, RT-qPCR, SYBR Green, gastroenteritis

## Abstract

**Simple Summary:**

Dogs often suffer from diarrhea caused by infectious agents, but not all of them are well studied. One of these agents, called Canine Kobuvirus (CaKoV), has been reported in several countries but has never been investigated in Ecuador. In this study, we looked for this virus in 250 dogs (200 with diarrhea and 50 apparently healthy) from different provinces of Ecuador. To do this, we created a new laboratory sensitive test that can detect even very small amounts of the virus with high precision and does not confuse it with other common dog viruses such as parvovirus or coronavirus. We found that 36.4% of the dogs were infected with CaKoV, especially in the province of Pichincha. Furthermore, a significant proportion of infected canines were found to be concurrently afflicted with other viral infections, thereby indicating that the presence of multiple infections may exacerbate the severity of the disease. This study reports the first molecular detection of CaKoV in Ecuadorian dogs with gastroenteritis, contributing to the current understanding of its geographic distribution and molecular epidemiology. Moreover, the insights derived from this research will underpin future studies that seek to elucidate the viral transmission dynamics and develop effective control measures.

**Abstract:**

Canine Kobuvirus (CaKoV) has been identified as an agent involved in gastrointestinal diseases among dogs worldwide, with a particular impact on young individuals. This study reports the first molecular detection of CaKoV in Ecuadorian dogs with gastroenteritis, using a sensitive SYBR Green-based RT-qPCR assay. The assay demonstrated high sensitivity, with detection limits approaching a single copy of genetic material (1 copy/μL), with a standard efficiency curve of 100.6% and a correlation coefficient of 0.997, facilitating accurate CaKoV detection even at a minimal number of gene copies; it was also highly specific for CaKoV genome amplification, as no amplification was shown for other canine enteric viruses [Canine Parvovirus (CPV-2), Canine Astrovirus (CaAstV), Canine Coronavirus (CCoV), and Canine Distemper Virus (CDV)], and demonstrated strong reproducibility across different runs. A total of 250 fecal samples were used to validate the assay and detect the presence of CaKoV, with 91 samples testing positive for CaKoV, confirming the virus’ presence across multiple provinces in Ecuador, with Pichincha reporting the highest number of positive samples. Phylogenetic analysis of the partial 3D gene sequence exhibited a nucleotide identity ≥ 90% with sequences of CaKoV strains from different countries around the world. Co-infections with other enteric viruses such as CPV-2, CCoV, and CaAstV were observed in 61.3% of CaKoV-positive samples from dogs with gastroenteritis, with triple co-infections (CPV-2, CaAstV, and CaKoV) being the most frequently detected combination in the study. The present study showed that CaKoV is circulating in domestic dogs affected with gastroenteric disease and in apparently healthy dogs. This work establishes CaKoV as a possible contributor to canine gastroenteritis in Ecuador, in addition to the typical viruses such as CPV-2 and CCoV; moreover, this study illustrates a molecular assay that is both rapid and reliable for the diagnosis of CaKoV.

## 1. Introduction

Canine gastroenteric diseases can be caused by different pathogens like viruses, bacteria, and parasites [1,2,3]. These diseases present a significant health concern for the global canine population, affecting the gastrointestinal tract, resulting in signs such as diarrhea, dehydration, vomiting, and, in severe cases, death [4]. These clinical signs can be associated with Canine Parvovirus 2 (CPV-2) [5], Canine Astrovirus (CaAstV) [6], Canine Coronavirus (CCoV) [7], Canine Distemper Virus (CDV) [8], Canine Kobuvirus (CaKoV) [4], and other pathogens. The canine industry has been economically affected by viral infections such as CDV and CPV-2, which can cause mortality rates ranging from 50 to 100% in unvaccinated populations [9]. Although CaKoV has not been associated with high mortality, its role as a co-infecting agent may contribute to disease severity and diagnostic challenges.

CaKoV is a newly discovered member in the *Picornaviridae* family, detected for the first time in the USA in 2011, corresponding to an Aichivirus A [4]. Aichivirus A encompasses six officially recognized viruses: Human Aichivirus, Canine Kobuvirus, Murine Kobuvirus, Kathmanadu sewage Kobuvirus, Roller Kobuvirus, and Feline Kobuvirus [10]. CaKoV belongs to the Kobuvirus Aichi species and the virus is also known as Aichivirus A2 [11]. Initially Aichivirus A was the only recognized member and was first detected in children with diarrhea in Japan [12].

CaKoV is a non-enveloped virus with an icosahedral shape and a single-stranded RNA genome of positive polarity [13]. Its genome spans roughly 8.1 to 8.2 kilobases and includes a single open reading frame (ORF) that encodes a polyprotein composed of about 2442 to 2475 amino acids. This precursor protein is subsequently cleaved by viral proteases into three structural components (VP0, VP3, and VP1) and seven non-structural proteins: 2A, 2B, 2C, 3A, 3B, 3C, and 3D [14]. The antigenic characteristics of CaKoV are mainly influenced by the capsid proteins, where VP1 represents the most variable and immunodominant region, while 3D and 2B are among the most conserved regions within the genome [15]. This virus is also found in the cerebellum, lungs, tonsils, and liver, suggesting that this virus has the potential to cause severe infections [16]. CaKoV infection frequently manifests with diarrhea and other clinical indicators like vomiting, anorexia, lethargy, abdominal pain, and dehydration, although it can also occur without any noticeable signs [17].

A recent Korean study found that this virus often occurs alongside several other viruses, including Canine Herpes Virus (CaHV-1), CDV, CPV-2, and both types of Canine Adenovirus (CadV-1 and CadV-2) [18]. For instance, mixed infections of CaKoV with CDV are the most frequently present, sometimes in combination with CPV-2 or CadV (1-2) [19].

CaKoV has been detected using molecular methods, with RT-PCR assays being the most commonly used [16,20,21]. Molecular methods such as RT-PCR and RT-qPCR are increasingly replacing conventional diagnostics for COVID-19 (virus isolations and antigen or antibody detection) as gold standard for virus detection, due to their superior specificity and sensitivity [22]. These molecular assays can offer near-complete accuracy in detecting viral genetic material [23]. Among molecular techniques, RT-PCR, in particular, has proven to be a highly sensitive and specific tool for identifying viral RNA, making it invaluable for both diagnosis and epidemiological surveillance of viral infections [24].

Despite the global prevalence of CaKoV, there is no data about its presence and distribution in Ecuador, primarily due to insufficient knowledge about virus epidemiology. However, based on epidemiological evidence, symptomatology, and reported mortality from various countries including China [25], Thailand [26], Vietnam [27], Brazil [18], Korea [19], Türkiye [28], Iran [29], Italy [30], the United Kingdom [31], Tanzania [17], and Japan [32], this virus demonstrates considerable etiological potential in gastroenteric diseases within this region. The current lack of CaKoV detection leads to misdiagnosis, ineffective treatments, and ongoing transmission of the virus within the canine population.

The undefined data complicates the understanding of acute gastroenteritis dynamics, which poses a particular risk to puppies and young dogs. Although no specific antiviral treatment is available for CaKoV, clinical management in veterinary medicine remains supportive, emphasizing rehydration and the prevention of secondary infections. This highlights the need for epidemiological and diagnostic studies to guide more targeted prevention and control strategies. The absence of CaKoV detection in Ecuador has delayed its recognition and may have allowed unnoticed circulation, highlighting the importance of establishing its presence as a first step toward future studies on pathogenesis, immunity, and prevention. The aim of this study is to fill this gap by reporting the first molecular detection and genetic characterization of CaKoV in Ecuadorian dogs through the standardization of a sensitive SYBR Green-based RT-qPCR assay and the phylogenetic analysis of the partial 3D gene, thereby contributing to the understanding of its molecular diversity and geographic distribution.

## 2. Materials and Methods

### 2.1. Sampling

For this study, 200 fecal samples were collected from dogs with gastroenteritis, presenting signs such as diarrhea, vomiting, depression, somnolence, and dehydration. These samples came from different provinces of Ecuador: Pichincha (147), Chimborazo (4), Imbabura (14), Santo Domingo (6), Guayas (15), and Tulcán (14). Additionally, 50 samples were also collected from apparently healthy dogs from a shelter in Pichincha, as a control group for the sick animals. All of them were kept under an adequate temperature of 4 °C and sent to the research laboratories of the Universidad de Las Américas (UDLA) for canine enteric virus examination (Supplementary Material). The analyzed samples had been previously tested for the presence of CPV-2, CCoV, and CaAstV using molecular techniques previously described, including a qPCR method with TaqMan probes as a method of comparison and contrast [21]. These same specimens were later reanalyzed to detect CaKoV through the RT-qPCR assay described in this study. This process enabled the assay’s standardization and validation, making it possible to identify CaKoV for the first time in co-infection with other canine enteric viruses within the tested samples. All experimental procedures were performed following the ethical and technical regulations of the Committee for the Care and Use of Laboratory and Domestic Animals of the Ecuadorian Agency for Phytosanitary and Animal Health Regulation and Control (AGROCALIDAD), under authorization number #INT/DA/019.

### 2.2. Nucleic Acid Extraction

Nucleic acid extraction was carried out by suspending the samples in 0.1 M phosphate-buffered saline (PBS) at a 1:1 ratio, in order to preserve the integrity of the biological material. The suspension underwent a thermal shock protocol to facilitate cell lysis, consisting of incubation in water-bath at 56 °C for 1 min, followed by 10 s of vortex, and then immediate freezing at −80 °C for 10 min; this cycle was repeated 3 times. After that procedure, the suspension was centrifuged at 12,000 rcf for 30 min at 4 °C. An aliquot of 250 μL of the supernatant was used for the extraction of viral RNA using TRIzol Reagent (Invitrogen by Life Technologies, Carlsbad, CA, USA) according to the manufacturer’s instructions. The phenol–chloroform method [33] was used for DNA extraction of the CPV-2 positive control to check the specificity of the assay. An internal control was included to ensure the integrity of the samples extracted based on the *B-actin* gene [34].

### 2.3. Primer Design

**Figure 1:**
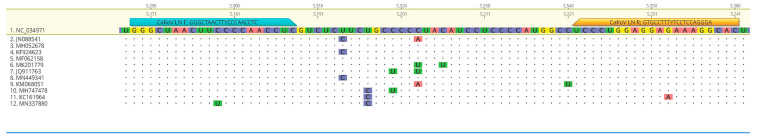
Nucleotide alignment used to design the primers described in Table 1.

Primers for the detection of CaKoV were designed based on conserved regions of the viral genome (Table 1). Reference sequences of complete genome of CaKoV were retrieved from the NCBI GenBank database (JN088541.1, MH052678.1, KF924623.1, MF062158.1, NC_034971.1, MK201779.1, JQ911763.1, MN449341.1, KM068051.1, MH747478.1, KC161964.1, and MN337880.1) and aligned (Figure 1) using the Geneious Prime^®^ 2022.2.2 program (https://www.geneious.com) to amplify a conserved region of the 2B gene (Table 1). Primer design parameters included a melting temperature (Tm) of approximately 59–60 °C and a GC content of 50–52%, ensuring optimal amplification under SYBR Green conditions. The exact nucleotide positions were not specified because the alignment was based on multiple complete genomes, and minor variations may occur among different isolates.

### 2.4. Standard Curve Construction

A synthetic double-stranded DNA fragment (gBlock Gene Fragments, IDT, Coralville, IA, USA) [35] containing the viral target region was employed to generate the standard curve. The customized gBlock was reconstituted in 50 μL of UltraPure™ DNase/RNase-Free Distilled Water (IDT) according to the manufacturer’s recommendations. Its concentration was then measured using a NanoDrop™ 2000 spectrophotometer (Thermo Fisher Scientific, Wilmington, DE 19810 USA). The quantified DNA value was entered into the DNA Copy Number and Dilution Calculator (Thermo Fisher Scientific) to calculate the precise amount of gBlock required to prepare an initial standard solution containing 10^8^ copies of the target sequence. Subsequently, a series of tenfold serial dilutions was prepared down to a final concentration equivalent to a single copy, forming the basis for the standard curve. To determine the assay’s analytical sensitivity (specifically, the limit of detection (LOD) and limit of quantification (LoQ), ten serial dilutions of the same gBlock were analyzed. The lowest concentration capable of being successfully amplified defined the detection and quantification thresholds of the developed RT-qPCR method, with the LOD representing the overall sensitivity of the assay.

### 2.5. RT-qPCR Assay

A two-step RT-qPCR assay was employed for CaKoV detection. RNA extracted from fecal samples was reverse-transcribed into cDNA using SuperScript III Reverse Transcriptase (Invitrogen™ Van Allen Way, Carlsbad, CA, USA) according to the manufacturer’s instructions. The cDNA was subjected to qPCR with a 10 µL of reaction mixture, containing 2X of PowerUp SYBR Green Master Mix (Applied Biosystems by Thermo Fisher Scientific), 1 µL of UltraPure™ DNase/RNase-Free Distilled Water (Invitrogen), 0.5 µM of each of the primers reported in Table 1 (final molar concentration), and 2 µL of extracted cDNA. Thermocycling conditions were optimized for Fast mode, which included the following parameters: UDG activation phase at 50 °C for 2 min, followed by enzyme activation at 95 °C for 2 min, succeeded by 40 cycles, with each cycle involving a brief denaturation at 95 °C for 3 s and a combined annealing and extension phase at 60 °C for 30 s, resulting in a run time of 1 h and 39 min (including the melting curve). The gBlock designed was used as the positive control, ddH_2_O as the negative control and non-template control (NTC) for each assay to ensure functionality. All reactions were performed in duplicate, and melting curves were analyzed to confirm product specificity.

### 2.6. Analytic Specificity of the RT-qPCR Assay

To determine the specificity of the RT-qPCR assay, positive controls for CDV, CPV-2, CaAstV, and CCoV were used. These were obtained from clinical sample specimens of dogs previously diagnosed with single infections by each respective virus and confirmed by sequencing. All control samples were subjected to the RT-qPCR protocol developed in this study using the previously established conditions (RT-qPCR assay section).

### 2.7. Analytic Repeatability of the RT-qPCR Assay

To evaluate the repeatability, reproducibility, and stability of the RT-qPCR assay, tenfold serial dilutions of the standard curve corresponding to 10^8^ and 10^4^ DNA copies were prepared, aliquoted, and stored at −20 °C until further analysis. The mean quantification cycle (Cq) values and the coefficient of variation (CV) obtained from the RT-qPCR runs were used to determine the assay’s precision and stability. The stability of the method was assessed by comparing CV values in both intra-assay and inter-assay analyses. For inter-assay repeatability, an aliquot from each of the five tenfold dilutions described above was independently amplified five times under identical reaction conditions. In each run, a fresh aliquot was thawed, and fluctuations in Cq values ranging between 0.5 and 1 cycle were examined to detect variability at each point of the curve. For intra-assay repeatability, five tenfold dilutions of the synthetic double-stranded DNA fragment containing the CaKoV target sequence were tested, performing five replicates for each dilution in a single RT-qPCR experiment. All experimental procedures adhered strictly to the MIQE 2.0 (Minimum Information for Publication of Quantitative Real-Time PCR Experiments) guidelines to ensure methodological transparency and analytical reliability [36].

### 2.8. Sequencing and Phylogenetic Analysis

End point PCR was performed using primers previously reported by Wang et al. 2020 [14] and listed in Table 1, to obtain a 504 bp amplicon of the 3D gene. PCR was carried using 0.5 µL of Buffer 10X, 50 mM of MgCl_2_, 0.5 µL of dNTPs 10 mM, 0.5 µM of each primer described in Table 1, 1 U of Platinum Taq DNA polymerase (Invitrogen by Thermo Fisher Scientific), and 2.5 µL of cDNA. Amplicons were sequenced using Sanger technology with BigDye Terminator v3.1 Cycle Sequencing Kit (Thermo Fisher Scientific, Vilnius, Lithuania) reagent and carried in the 3500 Series Genetic Analyzer (Applied Biosystems, Foster City, CA 94404, USA.) equipment. Electropherograms were edited and mapped based on a reference sequence obtained from the NCBI GenBank database, using Geneious Prime 2025.0.2 software package version 10.2.3 (https://www.geneious.com). The obtained sequences were aligned individually with other CaKoV sequences using Multiple Sequence Alignment Clustal Omega (https://www.ebi.ac.uk/jdispatcher/msa/clustalo, accessed on 29 September 2025) and similarities of nucleotides were inferred in Geneious Prime 2025.0.2 software package (https://www.geneious.com). A phylogenetic tree was generated using the Neighbor-Joining method with a Jukes–Cantor distance model to confirm the molecular identity and clustering of Ecuadorian CaKoV sequences with reference strains, recognizing that this approach was not intended for evolutionary inference; a phylogeny test bootstrap model with 1000 replicates and a threshold value of 75% that was integrated into the Geneious Prime 2025.0.2 software package (https://www.geneious.com) was used.

### 2.9. Diagnostic Performance of the RT-qPCR Assay

The diagnostic assay proposed in this research was validated according to Stage 1 guidelines of the WOAH Terrestrial Manual (Chapter 1.01.06). Analytical sensitivity was determined using a synthetic gBlock standard, and diagnostic parameters (sensitivity, specificity, PPV, and NPV) were calculated by comparing RT-qPCR results with previously characterized positive and negative control samples confirmed by RT-PCR. These indicators were computed using standard epidemiological formulas described by Fletcher (*Clinical Epidemiology*, Chapter 6) [37]. To evaluate the reliability of the RT-qPCR assay, key diagnostic performance parameters were calculated using standard epidemiological formulas. Diagnostic sensitivity was determined as TP/(TP + FN), while specificity was expressed as TN/(TN + FP). The positive predictive value (PPV) was computed using the formula [sensitivity × prevalence]/{[sensitivity × prevalence] + [(1 − specificity) × (1 − prevalence)]}. Similarly, the negative predictive value (NPV) was obtained as [specificity × (1 − prevalence)]/{[specificity × (1 − prevalence)] + [(1 − sensitivity) × prevalence]}. These calculations followed the guidelines described in Chapter 6 of Fletcher’s *Clinical Epidemiology* [38]. In these expressions, TP, FN, TN, and FP represent true positives, false negatives, true negatives, and false positives, respectively.

### 2.10. Statistical Analysis

A descriptive analysis of the evaluated samples was carried out; this includes the discrimination of individuals by age, considering juvenile puppies and adults or seniors according to the parameters previously indicated in 2021 for general canines [38]. The dataset included variables such as the number of viral copies, age of dogs, breed, province, and RT-qPCR diagnostic results of CaKoV, which were previously subjected to a Shapiro–Wilk test with a significance level of 0.05, to verify the data distribution. Pearson correlation coefficients were calculated to determine the relationship between quantitative variables (viral copies and age) and RT-qPCR results. Chi-square tests were performed to evaluate the association between categorical variables (breed and province) and RT-qPCR results. The detection of CaKoV was analyzed using a heatmap to visualize the differences between the positive and negative samples of each province. Additionally, Analysis of Variance (ANOVA) was conducted to assess the significance of age, breed, and province on RT-qPCR results. Additionally, Cohen’s Kappa test was performed between the results of the RT-qPCR assay based on SYBR Green developed in the present study and the one used as a comparison based on TaqMAN hydrolysis probes [21]. All statistical analyses were conducted using R software (version 4.4.0). These parametric tests were considered appropriate due to their robustness for moderately non-normal datasets and the quantitative nature of the variables analyzed.

### 2.11. GenBank Accession Numbers

The sequences obtained here of a part of the 3D gene were submitted to Genbank under the following accession numbers: 854 UDLA (PV848117); (PV848122); 843 UDLA (PV848123); 850 UDLA (PV848124); 858 UDLA (PV848125); 865 UDLA (PV848126); 867 UDLA (PV848127); 862 UDLA (PV848128); 864 UDLA (PV848129); 839 UDLA (PV848130); 770 UDLA (PV848131); 870 UDLA (PV848132); 869 UDLA (PV848133); 765 UDLA (PV848134); 848 UDLA (PV848135); 868 UDLA 851 UDLA (PV848118); 841 UDLA (PV848119); 863 UDLA (PV848120); 853 UDLA (PV848121); 866 UDLA (PV848136); 845 UDLA (PV848137). The sequences obtained in the present study which showed 100% nucleotide identity between them were not sent to GenBank.

## 3. Results

### 3.1. Analytical and Diagnostic Validation Parameters

#### 3.1.1. Standard Curve and Sensitivity

**Figure 2:**
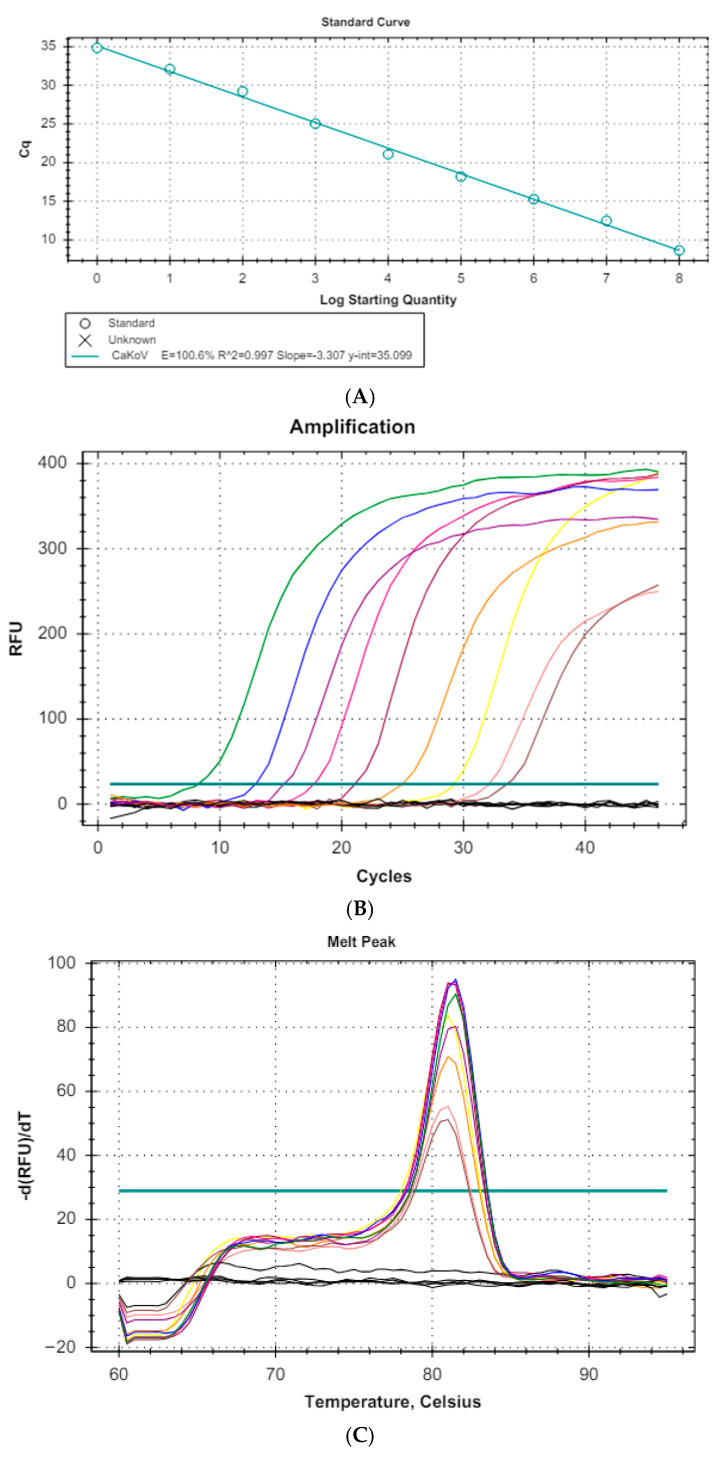
Assay to determine the standard curve of CaKoV. (**A**) Standard curve. (**B**) Amplification plot. (**C**) Melting curve.

A ten-fold serial dilution of the gBlock containing the target sequence of CaKoV produced a standard curve with an efficiency of 100.6%, a correlation coefficient of 0.997, and a slope −3.307 (Figure 2A). Each sample’s amplification plot (Figure 2B) allowed the identification of LoD and LoQ, indicating that the limit of detection (LoD) was established at 10^0^ copies/µL of genetic material. All amplified samples exhibited a melting curve at 81 °C, which matched the target fragment (Figure 2C). No negative controls, dimers, or non-specific products were detected in any run.

#### 3.1.2. Analysis of Analytic Specificity

The RT-qPCR assay amplified CaKoV, confirming its presence as the possible etiological agent of gastroenteritis in dogs. Positives controls for CDV, CPV-2, CaAstV, and CCoV showed no cross-amplification, highlighting the assay’s high specificity for CaKoV.

#### 3.1.3. Diagnostic Performance of the Assay

Based on the performance metrics calculated for the evaluated diagnostic method, a sensitivity of 100.0% was observed, indicating that all positive cases were correctly identified. The specificity reached 93.7%, reflecting a high ability to correctly classify negative samples. Furthermore, the negative predictive value (NPV) was 100.0%, demonstrating that negative results are highly reliable, while the positive predictive value (PPV) was 90.1%, confirming a strong probability that positive results truly correspond to infected individuals. These findings highlight the excellent diagnostic performance of the assay, both for confirming the presence of the pathogen and for reliably excluding its absence.

#### 3.1.4. Analysis of Repeatability

The assessment of repeatability conducted with gBlock dilutions from 10^8^to 10^4^ copies exhibited an inter-assay coefficient of variation ranging from 0.305% to 0.876% and an intra-assay coefficient of variation ranging from 0.335% to 0.948% (Table 2).

### 3.2. Detection of CaKoV

The test identified the presence of CaKoV in 80 of the samples from dogs with gastroenteritis, corresponding to 40% of the samples analyzed in this group. On the other hand, of the apparently healthy dogs, only 11 samples tested positive, representing 22% of the samples in this group. Of the total samples analyzed, CaKoV was detected in 36.4% of cases. The calculation of Cohen’s Kappa index resulted in 0.84, indicating agreement between the results of the two methods, with 10 additional samples for the SYBR Green-based method with fewer than 100 gene copies (Supplementary Material).

#### 3.2.1. CaKoV Distribution in Dogs by Location 

**Figure 3:**
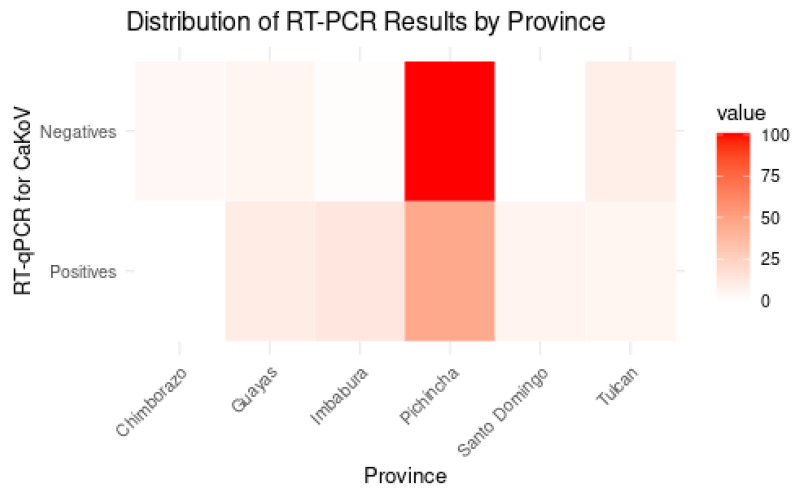
Heatmap illustrating the distribution (absolute values) of CaKoV-positive and -negative samples in dogs with gastroenteritis across Ecuadorian provinces. Color intensity represents the absolute number of CaKoV-positive samples, with darker red shades indicating higher case counts. Due to sampling imbalance between provinces, the figure provides a descriptive overview and does not reflect normalized prevalence.

The Shapiro–Wilk test indicated that the dataset does not follow a normal distribution (*p*-value < 0.05). The analysis was conducted across seven provinces in Ecuador, with Pichincha having the highest number of positive cases (46 samples in dogs with gastroenteritis and 11 samples in apparently healthy dogs), followed by Imbabura (13), Guayas (10), Santo Domingo (6), and Tulcán (5); Chimborazo did not present any positive samples (Figure 3). Although the analysis included seven provinces, no statistically significant differences were detected among them (Chi-square test, *p* > 0.05).

#### 3.2.2. CaKoV Distribution in Dogs by Age 

The distribution of RT-qPCR results for CaKoV detection in dogs with gastroenteritis by age group revealed the highest presence of virus in puppies with 64 positives out of 140 samples and an average number of gene copies of 3.40 × 10^5^. Adults showed 16 positives out of 53 samples, with a slightly higher average number of gene copies of 3.82 × 10^5^ (Table 3). Senior dogs had no positive samples among the seven tested animals, and no viral copies were detected in this group, indicating a higher prevalence and number of gene copies of CaKoV in younger dogs. In apparently healthy dogs, CaKoV was detected in 11 adults with an average load of 295.

#### 3.2.3. Co-Infections

Of the 80 samples from dogs affected with gastroenteritis that tested positive for CaKoV, co-infections with other enteric viruses were frequently observed. The most common co-infection involved CaKoV, CaAstV, and CPV-2, accounting for almost half (48.75%) of the positive samples. Dual infections were less common, with CaKoV/CaAstV being detected in 20 samples, and CaKoV/CPV-2 in eight samples. Notably, no samples showed co-infection with CaKoV and CCoV alone. Co-infections involving three or four viruses were rare, with only two samples testing positive for all four viruses (CaKoV, CaAstV, CPV-2, and CCoV) (Table 4). CPV-2 alone was found in 5 of the 11 samples from asymptomatic dogs that tested positive for CaKoV, and the same virus (CPV-2) was found in 15 of the samples that tested negative for CaKoV (Supplementary Material).

#### 3.2.4. Statistics

The Pearson correlation analysis indicated a weak correlation for both ‘Gene copies’ and ‘Age’ with CaKoV detection results (positive/negative), with coefficients of −0.0930 and 0.1695, respectively, indicating that there is insufficient evidence to establish a statistical association between these factors and RT-qPCR results. In contrast, the ANOVA assay indicated a significant effect of both ‘Age’ and ‘Province’ on CaKoV detection, with *p*-values of 0.0181 and <0.001, respectively; conversely, ‘Breed’ (Supplementary Material) did not show a significant impact on positive RT-qPCR results (*p*-value: 0.531), indicating that this factor may not play a substantial role in the presence of CaKoV.

### 3.3. Sequencing and Phylogenetic Analysis

**Figure 4:**
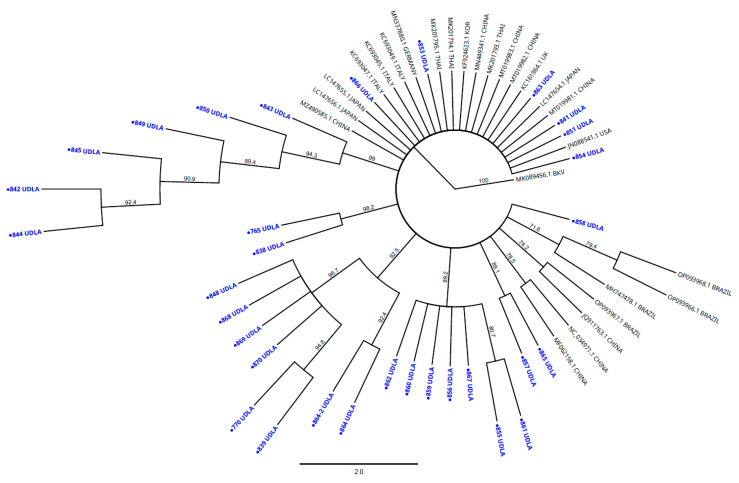
The phylogenetic analyses were performed using sequences obtained from a portion of 3D gen in the present study and other CaKoV sequences from other countries present in GenBank. Sequences were aligned using Multiple Sequence Alignment Clustal Omega included in the Geneious Prime 2025.0.2 software package (https://www.geneious.com). The phylogenetic tree was built in the Geneious Prime 2025.0.2 software package (https://www.geneious.com). Numbers along the branches refer to bootstrap values for 1000 replicates. The scale bar represents the number of substitutions per site. Bovine Kobuvirus (BKV) was used as the outgroup. CaKoV sequences in blue and marked with circles were obtained from the Ecuadorian dogs affected with gastroenteritis.

The Ecuadorian CaKoV sequences (*n* = 32), obtained from a 3D fragment, showed high NT similarity among themselves, ranging from 94.65% to 100%. The sequences studied also showed high NT similarity with other sequences from various countries retrieved from GenBank, specifically, an NT similarity between 94.06 and 97.43% with sequences from Brazil; 94.06–97.82% with sequences from China; 93.47–96.44% with sequences from Germany; 95.01–97.05% with sequences from Italy; 95.04–97.62% with sequences from Japan; 94.26–97.62% with sequences from South Korea; 94.26–97.03% with sequences from Thailand; 93.27–95.84% with sequences from the United States; and 94.06–95.84% with sequences from the United Kingdom. The NT matrix showed high similarity between the sequences described above, showing less than 7% of NT differences between them (Supplementary Material). The phylogenetic analysis of a portion of the 3D region showed that the sequences obtained here clustered with other sequences of CaKoV from Brazil, Germany, the United States, the United Kingdom, Thailand, Japan, China, Italy, and South Korea. All sequences were clustered in a unique group (clade bootstrap value 100%) (Figure 4).

## 4. Discussion

Canine gastroenteritis is a disease attributable to various factors, including bacteria [3], parasites [2], and viral agents which are frequently identified as primary causes [39]; it affects the gastrointestinal tract of dogs. Rapid and accurate detection of viral pathogens is crucial to prevent severe complications, allowing the identification of the exact cause and appropriate treatment [24]. The qPCR technique offers high accuracy in pathogen detection and the quantification of gene copies, which is essential for monitoring infections. The assay validated in this study shows high repeatability (Table 2), due to its precision and robustness in each repetition using the same samples [36].

The current assay, validated according to the WOAH Terrestrial Manual guidelines, demonstrated excellent analytical parameters, including very low detection limits (LoQ and LoD), being capable of detecting as few as 10^0^ copies of viral genetic material. For instance, in 2019, an assay based on end point PCR was executed for the simultaneous detection of CDV, CPV-2, and CaKoV, but it lacked the ability to quantify gene copies and was not validated under internationally recognized standards, reporting an LOD of 10^3^ copies [9]. Other studies performing RT-qPCR assays based on the SYBR Green method reported a single-plex assay for CaKoV in 2020 with an LoD of 10^1^ copies [20], and a duplex assay with CaAstV with the same LoD [16]. A multiplex TaqMan RT-qPCR assay to detect four canine enteric viruses was executed in 2020, exhibiting an LoD of 10^2^ copies [21]; however, there is no evidence that these methods underwent a complete analytical and diagnostic validation process as recommended by WOAH, which limits their comparability and reliability for routine use. Considering that TaqMan hydrolysis probe-based assays are expensive in relation with SYBR Green [40], the present method presents a cost-effective and reliable alternative for single-target diagnosis. Although probe-based assays generally reduce the risk of non-specific amplification, the melting curve analysis performed in this study confirmed that only one specific amplicon was generated. Furthermore, the complete analytical validation following WOAH guidelines demonstrated high specificity and repeatability, supporting that properly optimized SYBR Green assays can achieve comparable performance to probe-based assays.

This increased sensitivity obtained in the present study allows the detection of extremely low number of gene copies, which is essential for early-stage diagnosis and treatment, making this assay highly effective for clinical and research applications [41]. The ability to detect a low number of gene copies is particularly important because viruses can persist in a host with a low number of viral copies and no symptoms; however, in immunosuppressed organisms, such as those under stress conditions and affected by concurrent disease, these latent infections may become reactivated [42]. To the best of our knowledge, no commercial or hydrolysis probe-based RT-qPCR kits are currently available for CaKoV detection, precluding direct head-to-head comparison. Future studies should consider benchmarking our SYBR Green assay against hydrolysis probe-based protocols once these become available. The detection of a low number of CaKoV gene copies may reflect early or late stages of infection, variations in sample quality, or asymptomatic viral carriage [26]. Although these results do not demonstrate pathogenicity, they provide epidemiological evidence of virus circulation among dogs [27], emphasizing the importance of molecular surveillance for enteric viruses in Ecuador.

In South America, CaKoV has only been reported in Brazil [18], with Ecuador being the second country reporting the presence of this pathogen; the lack of information in other countries of South America, including bordering countries (Peru and Colombia), limits the understanding of the viral epidemiological distribution. This study identified the presence of CaKoV in 91 out of 250 samples, representing 36.4% of the analyzed specimens. In this study, we also included a control group of 50 clinically healthy dogs, of which 11 (22%) tested positive for CaKoV. The inclusion of healthy controls strengthens the epidemiological interpretation of our results and underscores the need for case–control and longitudinal studies to clarify the clinical significance of CaKoV infection in dogs. This detection rate aligns with findings from previous studies, such as those conducted by [43], where a prevalence rate of 50.46% was reported in canine populations in China, which emphasizes the presence of CaKoV as a possible etiological agent in canine gastroenteritis. However, studies performed in Asia, such as those in China [25] and Thailand [26], showed a significantly lower prevalence, with a CaKoV positivity of 4.7% and 17.59%, respectively. A study conducted in Brazil identified only 3 positives out of 53 samples, yielding an occurrence rate of 5.7% [44]. The circulation of different strains in different geographic areas may influence the distribution of the virus, with notable differences, as mentioned above; although several studies have proposed a relationship between intercontinental strains, no differences have been defined in terms of pathogenic and epidemiological effect [27,28,45]. In this study, a control group of 50 clinically healthy dogs is also included, of which 11 (22%) tested positive for CaKoV. This finding confirms the occurrence of subclinical infections and supports previous evidence that asymptomatic dogs contribute to viral maintenance in the Ecuadorian dog’s population [28,43,45]. Including healthy controls improves the epidemiological interpretation of our results and highlights the importance of future case–control and longitudinal studies to better understand the clinical and epidemiological behavior of CaKoV infection.

Several factors such as population density, veterinary practices, climatic conditions, and canine health might play a crucial role in the distribution of the virus in the canine population [39,46,47]. The regional analysis revealed significant geographical variations in CaKoV presence across seven Ecuadorian provinces, with Pichincha presenting the highest number of positives. However, Pichincha had the largest number of analyzed samples, which may have contributed to this rate; in order to obtain more representative and significant values, it would be beneficial to execute an epidemiological study [23,48]. The unequal sample sizes across provinces, particularly the overrepresentation of Pichincha, limit any epidemiological interpretation. As the aim of this study is not to establish regional prevalence but to report the first molecular detection of CaKoV in Ecuador, these results should be considered descriptive.

Dogs with gastroenteritis and an age range of 1–12 months showed the highest presence of CaKoV compared with age groups of adults and senior dogs, with 64/80 positives. This age-related distribution underscores the vulnerability of younger dogs to this pathogen, consistent with [29], who reported a frequency of CaKoV of 27% in young populations. The higher presence of CaKoV in this age group may be attributed to the immunological immaturity of puppies, particularly their lower level of circulating IgGs, which compromises their ability to perform an effective immune response [49]. In young dogs, vaccination provides immunological protection against viral pathogens [50]; however, there is currently no vaccine available for CaKoV, leaving this population vulnerable to infection. Although maternal-derived antibodies provide partial protection against several enteric viruses, there is currently no evidence of specific maternal immunity against CaKoV. The detection of CaKoV in apparently healthy dogs should not necessarily be interpreted as subclinical infection, since its low pathogenic potential and the presence of co-infections—particularly with CPV-2—suggest that these animals may act as carriers of the virus rather than showing active disease. In this assay, 22.5% of samples tested positive solely for CaKoV, in the absence of other viruses that were tested (CPV-2, CCoV, and CaAstV); since the disease is present and CaKoV is the only detected pathogen, it may contribute to the disease’s exacerbation [4,18,30,51] or hinder accurate diagnosis, as it has not been considered a causative agent of gastroenteric disease in dogs in Ecuador. Therefore, it is imperative to incorporate diagnostic protocols for this pathogen to improve veterinary practices in Ecuador.

In this study, the most common combination in sick dogs was the triple co-infection of CaKoV, CaAstV, and CPV-2, found in 39 of the 80 CaKoV-positive samples (48.75%). This contrasts with findings in other studies such as the one performed in China in 2020, where all the CaKoV-positive samples were positive for CPV-2, only one sample tested positive for the co-infection of CaKoV and CaAstV, and no co-infection was detected with CaKoV and CCoV [14]. Similar co-infection patterns have been reported elsewhere [16]. This comparison illustrates that, although CaKoV is commonly detected together with other enteric viruses, especially in young dogs, the frequency and combinations observed may vary between studies, likely due to methodological and local epidemiological factors. In contrast, another study performed a duplex assay for the detection of CaKoV and CaAstV and reported only 1 positive co-infection out of 48 samples analyzed, whereas this study reported 20 samples positive for this co-infection [16]. A study performed in China reported the presence of CaKoV and CCoV co-infection in five (9.8%) samples of dogs presenting diarrhea [52]. The third most common co-infection involved CaKoV and CPV-2 alone, which was present in 8 samples positive for CaKoV, but CPV-2 was present in 61.25% of the total analyzed samples, making it comparable to the results reported in Brazil [44] where the presence of CaKoV was detected in 3/53 samples, and all CaKoV positive samples were also co-infected with CPV-2, although in that study this specific combination was observed exclusively. Some samples that tested negative for CaKoV were positive for the other enteric viruses that were analyzed (CPV-2, CCoV, and CaAstV), which are considered potential causative agents of gastroenteritis in dogs (Supplementary Material); however, there were samples that did not amplify for any of the four analyzed viruses, despite the fact that all the dogs examined presented gastroenteritis disease, suggesting that other, uncharacterized pathogens may be involved in causing the disease [2,3,9,53]. Future studies identifying the most common pathogens related with gastroenteric disease in Ecuadorian dogs must be conducted in order to develop a routine method capable of providing a correct and rapid diagnosis of this disease, thus improving the clinical attention in Ecuadorian veterinaries and reducing mortality in the canine population related with misdiagnosis and incorrect treatments [41,54,55].

The phylogenetic analysis carried out by sequencing a fragment of the 3D region showed the presence of the virus in dogs from Ecuador. Nucleotide identities >90% were observed between our 3D sequences and those from Brazil, Germany, the United States, the United Kingdom, Thailand, Japan, China, Italy, and South Korea, which were previously deposited in GenBank (Figure 3). It is necessary to carry out subsequent studies that analyze the most variable regions of this virus, such as VP1 or VP3, as well as the complete genome to identify the circulating lineages and their direct interaction with other viruses that could be generating a more serious disease [14]. Because the role of the virus in cases of gastroenteritis is still controversial, there are no preventive measures, such as vaccines or specific treatments. This could be facilitating its circulation and mutagenesis, thus increasing the likelihood that it will acquire a more relevant role in the etiology of the disease. Recent outbreaks and identifications of emerging viruses draw attention to the poor management of epidemiological containment of canine enteric viruses in Ecuador [56,57,58], which could lead to increases in morbidity and mortality rates, underscoring the urgent need for improved surveillance and prevention strategies.

## 5. Conclusions

This study represents the first molecular detection of CaKoV in dogs from Ecuador, documenting its circulation in animals with gastroenteritis and in apparently healthy individuals. The RT-qPCR assay developed here demonstrated high sensitivity and specificity, providing a reliable and cost-effective diagnostic tool for CaKoV detection and surveillance. Although CaKoV was frequently found in co-infection with other enteric viruses, its detection contributes baseline data for understanding its distribution rather than proving a direct etiological role in gastroenteric disease. These findings underscore the importance of continuous molecular monitoring and further case–control and pathogenicity studies to better define the clinical relevance and genetic diversity of CaKoV circulating in Ecuadorian dogs.

## Figures and Tables

**Table 1:** Primers used in this study.

Primers	Target	Sequences	Assay	Length	Reference
CaKoV LN F	2B gene	5′GGGCTAACTTYCCCAACCTC 3′	RT-qPCR	73 bp	This Study
CaKoV LN R		5′GTGCCTTTYTCCTCCAGGGA 3′			
CaKoV-3D-F CaKoV-3D-R	3D gene	5′CCCTGGAACACCCAAGGCCGCT 3′ 5′TCTGGTTGCCATAGATGTGGTG 3′	End point PCR	504 bp	[14]

**Table 2:** Repeatability assay using standard ten-fold serial dilutions.

Copy Number	Inter-Assay		Intra-Assay	
	Cq Mean	Cq Std Dev	Cq Mean	Cq Std Dev
10^8^	9.79	0.876	10.19	0.948
10^7^	15.32	0.413	14.83	0.550
10^6^	17.99	0.485	17.72	0.360
10^5^	21.11	0.324	20.88	0.412
10^4^	24.54	0.305	24.47	0.335

**Table 3:** RT-qPCR results for CaKoV detection by age group, presenting the number of positive and negative samples for virus detection, along with the average number of gene copies detected in each group. * = Each age range group, defined as follows: puppies (1–12 months), adults (13–84 months), and seniors (over 85 months).

Distribution of CaKoV in Gastroenteritis Dogs by Age				
Age Group	* Age Range (months)	Average Number of Gene Copies	Positives	Negatives
**Puppies**	1–12	3.40 × 10^5^	64 (32%)	76 (38%)
**Adults**	13–84	3.82 × 10^5^	16 (8%)	37(18.5%)
**Seniors**	85+	0	0 (0%))	7(3.5%)

**Table 4:** Distribution of CaKoV co-infections with CaAstV, CPV-2, and CCoV in CaKoV-positive samples from dogs with gastroenteritis.

CaKoV Co-Infections in Dogs with Gastroenteritis					
Combination N°	CaKoV	CaAstV	CPV-2	CCoV	Total
**1**	x	x			20 (25%)
**2**	x		x		8 (10%)
**3**	x			x	0 (0%)
**4**	x	x	x		39 (48.75%)
**5**	x		x	x	0 (0%)
**6**	x	x		x	2 (2.5%)
**7**	x	x	x	x	2 (2.5%)

## Data Availability

The original contributions presented in this study are included in the article/Supplementary Material. Further inquiries can be directed to the corresponding author.

## Supplementary Materials

The following supporting information can be downloaded at: https://www.mdpi.com/article/10.3390/vetsci12111076/s1, Sample information and CaKoV Nucleotide Matrix.

## Abbreviations

The following abbreviations are used in this manuscript:

CaKoV	Canine Kobuvirus
PCR	Polymerase Chain Reaction
RT-PCR	Reverse Transcription Polymerase Chain Reaction
UDLA	Universidad de las Americas
CPV	Canine Parvovirus
CaAstV	Canine Astrovirus
CDV	Canine Distemper Virus
ORF	Open Reading Frame
VP	Virulence Protein
NTC	Non-Template Control
LoD	Limit of Detection
LoQ	Limit of Quantification
NT	Nucleotides
N°	Number
CV	Coefficient of Variation
Cq	Cycle of Quantification
